# Carcinoma prostate masquerading as a hemorrhagic pelvic cyst

**DOI:** 10.1590/S1677-5538.IBJU.2015.0207

**Published:** 2017

**Authors:** Rajat Arora, Arun Jacob Philip George, Anu Eapen, Antony Devasia

**Affiliations:** 1 Department of Urology, Christian Medical College, Vellore, Tamil Nadu, India;; 2 Department of Urology, Christian Medical College, Vellore, Tamil Nadu, India

## CASE

A 53 year-old man, with untreated lower urinary tract symptoms for two years was catheterized for acute retention of urine. He had an enlarged, boggy non tender prostate.

**Figure 1:**
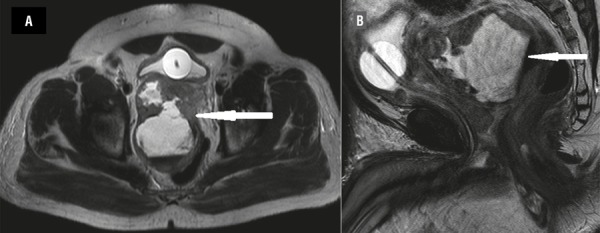
(A and B): Hemorrhagic cyst (arrow) at initial presentation.

The transrectal ultrasound (TRUS) revealed a fluid filled cystic mass arising from the prostate and 200cc of hemorrhagic fluid was aspirated. There was no growth on culture of fluid and Xpert® MTB/RIF along with three acid fast bacillus smears was negative for tuberculosis. Cytology was not performed on the aspirated fluid. Magnetic resonance imaging revealed prostate volume of 52cc and a 94x77mm cystic and solid lesion (Figure-1) with seminal vesicle and rectal infiltration. There was abnormal signal intensity with T2-weighted hypointensity of the peripheral zone of the prostate. Close to the apex of the prostate, the cystic lesion merged with the right side of the base of the prostate. Above mentioned area near the apex showed restricted diffusion. His prostate specific antigen (PSA) was 910.0ng/mL. TRUS guided biopsy of the cyst wall revealed adenocarcinoma of prostate (Gleason grade: 4+4=8). The Technetium-99m methylene diphosphonate scan showed no evidence of osseous metastasis.

**Figure 2:**
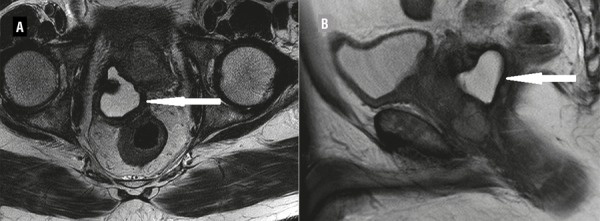
(A and B): Reduction in size of cyst (arrow) after androgen deprivation therapy.

Based on the locally advanced nature of the disease, he was initiated on neoadjuvant Leuprolide acetate 22.5mg subcutaneously (luteinizing hormone releasing hormone analog) and he voided successfully without a catheter on follow-up. At three months, his PSA was 21.9ng/mL with marked reduction in the size of the cystic lesion (50x40mm) (Figure-2). He received radiotherapy (Intensity Modulated Radiation Therapy technique with image guidance) after six months of androgen deprivation. A total dose of 79.2Gy was delivered in 44 fractions and his nadir PSA was 0.04ng/mL after 18 months of follow-up. Leuprolide is being continued for three years.

Hemorrhagic pelvic cyst remains a rare presentation of carcinoma prostate (1-5). With a raised PSA, a high index of suspicion should be maintained with a low threshold for a TRUS guided biopsy.
